# Exploring the Link between Head and Neck Cancer and the Elevated Risk of Acute Myocardial Infarction: A National Population-Based Cohort Study

**DOI:** 10.3390/cancers16101930

**Published:** 2024-05-18

**Authors:** Dong-Kyu Kim

**Affiliations:** 1 Department of Otorhinolaryngology-Head and Neck Surgery, Chuncheon Sacred Heart Hospital, Hallym University College of Medicine, Chuncheon 24253, Republic of Korea; entkim@hallym.or.kr; Tel.: +82-33-240-5180; Fax: +82-33-241-2909; 2 Institute of New Frontier Research, Division of Big Data and Artificial Intelligence, Hallym University College of Medicine, Chuncheon 24253, Republic of Korea

**Keywords:** cancer, head and neck, myocardial infarctions, cardiovascular, cohort, risk

## Abstract

**Simple Summary:**

The relationship between head and neck cancer and heart attack is a complex area of medical research that has potential implications for patient care and outcomes. This study aimed to assess the risk of developing heart disease attributable to head and neck cancer. In this study, we examined the association between head and neck cancer and the risk of heart attack using a database of 1,025,340 South Korean patients. There were no associations between the development of a heart attack in head and neck cancer survivors who did not receive radiation or chemotherapy. Our results consistently indicated a negligible long-term increase in the risk of developing a heart attack after a head and neck cancer diagnosis.

**Abstract:**

Enhanced screening protocols for cancer detection have increased survival in patients with head and neck cancer (HNC), which highlights the need to address the sequelae of therapy-induced cardiovascular complications. This study was conducted to assess the incidence and risk of acute myocardial infarction (AMI) in patients with HNC who have not undergone radiation or chemotherapy using a comprehensive, population-based cohort dataset. A total of 2976 individuals without cancer and 744 individuals with HNC were matched using the propensity score method. The findings indicated that the occurrence rates of AMI were comparable between the HNC (2.19) and non-cancer groups (2.39). Cox regression analysis did not demonstrate a significant increase in the risk of AMI in patients with HNC (hazard ratio: 0.93, 95% confidence interval: 0.50–1.73). No increased risk of AMI was observed in the HNC group compared to the non-cancer group, regardless of the time since the HNC diagnosis. Subgroup analyses showed no notable differences in the AMI risk between the groups when considering sex, age, comorbidities, and cancer type. This study showed that patients with HNC who have not been treated with radiation or chemotherapy did not exhibit an increased incidence or risk of AMI compared to individuals without cancer.

## 1. Introduction

Head and neck cancers (HNC) are a heterogeneous group of neoplasms originating in the oral cavity, oropharynx, nasopharynx, paranasal sinuses, hypopharynx, larynx, and salivary glands [1]. The histological types of HNC are primarily determined by the origin and nature of malignant cells, including squamous cell carcinoma, adenocarcinoma, sarcoma, lymphoma, melanoma, and neuroendocrine carcinomas [2]. The histological type of HNC plays a critical role in determining the treatment approach and prognosis. Squamous cell carcinomas, due to their prevalence, have been the focus of extensive research, leading to a more nuanced understanding of their behavior and best practices for treatment [3]. For other less common histologic types, treatment strategies are often based on the tissue of origin and the molecular characteristics of the tumors, as well as on the general principles of oncology. Because of its location, HNC can significantly affect breathing, swallowing, speaking, and appearance. HNC encompasses various cancers that originate in the head and neck region, each with unique histological (tissue-based) characteristics.

Risk factors for HNC include smoking and chewing tobacco, excessive alcohol consumption, viral infections, such as human papillomavirus and Epstein–Barr virus, radiation exposure, and occupational exposure [4]. Therapeutic interventions for HNC typically involve surgical resection, which may be utilized in isolation or in conjunction with adjuvant radiotherapy and concurrent chemoradiotherapy, delineating the cornerstone modalities for patient management in this domain. The advent of enhanced screening protocols for early cancer detection, along with the integration of multimodal treatment regimens, has augmented survival outcomes in individuals diagnosed with HNC [3]. This advancement has led to an increasing cohort of HNC survivors, thereby elevating the clinical imperative to address the sequelae of cancer therapy-induced cardiovascular complications, which significantly influence both morbidity and mortality rates in this patient population [5].

Hypertension (HTN), diabetes mellitus (DM), and dyslipidemia are established risk factors for ischemic vascular events, including stroke and acute myocardial infarction (AMI) [6,7]. Cohort studies focusing on HNC have also examined the incidence of stroke and AMI after HNC treatment. A population-based study revealed an elevated risk of stroke among patients with HNC, particularly in younger individuals and those undergoing combined radiotherapy and chemotherapy [8]. Another study on HNC reported that the risk of cardiovascular disease increased with radiation exposure after adjusting for other socioeconomic and clinical risk factors [9]. In addition, a recent population-based investigation elucidated that the prevalence of HTN and DM is significantly higher in individuals diagnosed with HNC than in control groups [10]. Even after adjusting for vascular risk factors, including DM, HTN, and dyslipidemia, the incidence rates of both stroke and AMI persisted at higher levels in the HNC cohort, indicating an intrinsic risk associated with HNC beyond the traditional vascular risk profiles [10].

Numerous investigations have shown that radiation therapy is linked to an elevated incidence of cerebrovascular disease among individuals diagnosed with HNC [11,12,13]. The pathophysiological underpinnings of radiation-induced injury to the carotid or cerebral arteries remain partially elucidated but are hypothesized to involve endothelial dysfunction, damage and blockage of the vasa vasorum, and expedited atherosclerosis. In addition, the incidence of thromboembolic events has been observed to escalate following the use of chemotherapy across a spectrum of cancer types. While the precise mechanisms underlying chemotherapy-induced thromboembolism remain to be fully defined, ischemic cerebrovascular accidents attributed to chemotherapy have been documented in several cancer types, including HNC, breast cancer, and lymphoma, which indicates a broader oncological impact [14,15,16]. Radiation and chemotherapy are known to be associated with an increased risk of developing ischemic cardiovascular diseases. Therefore, this study aimed to assess the risk of ischemic cardiovascular disease attributable directly to HNC, in addition to the effects of treatment modalities. To this end, we conducted an in-depth analysis of the incidence and future risk of AMI among patients with HNC using an expansive dataset that encompasses a wide array of diseases across a representative nationwide population. This dataset facilitated a thorough investigation of possible connections between HNC and cardiovascular health issues. Stringent controls were applied to mitigate the impact of potential confounders and ensure the integrity of the findings.

## 2. Materials and Methods

### 2.1. Characteristics of the Nationwide Population-Based Cohort

This comprehensive study draws on a vast longitudinal dataset from the Korean National Health Insurance Service, which was meticulously compiled from anonymized health claims data spanning over a decade. The dataset, which captures a broad swath of the population of over one million adults, or approximately 2.2% of South Korea’s population, shows exceptional stability with negligible attrition [17,18]. By deliberately excluding immigrants—a group that represents a small fraction of the population—the researchers ensured that the representativeness of the study was not adversely affected. Unique identification numbers assigned to South Koreans at birth eliminated any potential overlapping or missing data within this dataset. This dataset is further enriched with detailed records from healthcare providers, offering insights into the infrastructure of medical facilities, including the variety of services, personnel qualifications, and availability of medical equipment. By covering a comprehensive array of healthcare utilization metrics, such as mortality rates, hospital admissions, outpatient services, and prescription histories, this dataset forms a robust foundation for analyzing health trends and outcomes. Despite its proven reliability in previous verification studies, the reliance of the dataset on claims data for disease diagnosis presents a notable limitation, necessitating precise operational definitions to ensure the accuracy of health status representation. This study adhered to stringent ethical guidelines through meticulous de-identification of the data and safeguarding of participant confidentiality while reinforcing the integrity and reliability of its findings.

### 2.2. Retrospective Cohort Strategy

**Figure 1:**
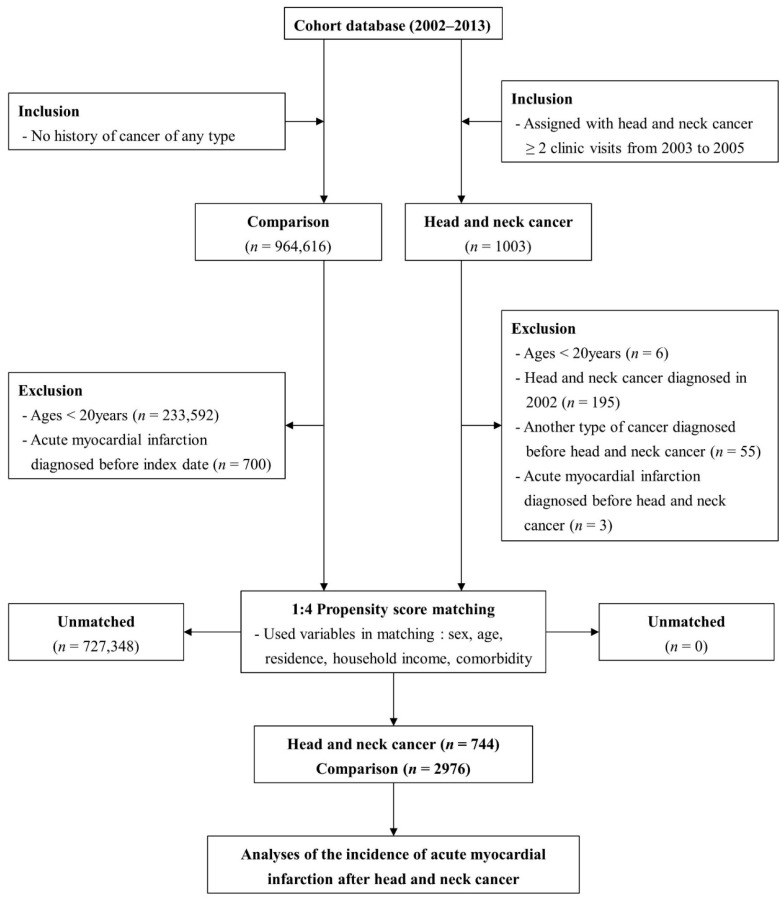
The process of participant selection in the study.

To examine the link between HNC and the incidence of AMI, we established two separate groups for comparison: a target group of individuals diagnosed with HNC and a control group without a cancer diagnosis. To eliminate the influence of cancer on the primary outcome measure, the control group was selected from participants who did not develop any type of cancer and matched with the target cohort group. During this matching procedure, sex, age, residence, income level, and comorbidities were identified as independent variables to achieve an equilibrium between the two cohort groups. This study, structured as a retrospective cohort analysis, compared the primary outcomes between these groups over time, using historical data gathered from existing records. The present study began at a defined point in the past and monitored events from that time until the current day. The process of participant selection is shown in Figure 1, with HNC patients identified during the base period of 2003–2005 using specific diagnostic codes for the different types of HNC. The classification of HNC within the cohort was delineated through a spectrum of sub-diagnostic codes, including malignancies of the oral cavity (C00–C06), neoplasms of the salivary gland (C07–C08), carcinomas of the oropharynx (C09–C10), malignancies of the nasopharynx (C11–C12), tumors of the hypopharynx (C13–C14), carcinomas of the paranasal sinuses (C30–C31), and malignancies of the larynx (C32). Conversely, the control group comprised individuals without cancer, chosen from a broader database population, and matched to the HNC cases on a 4:1 ratio using a propensity score approach that considered variables such as sex, age, geographical location, income, and pre-existing health conditions. Comorbid conditions were matched using the Charlson Comorbidity Index (CCI), a method frequently used in studies based on claims data [19]. The CCI is commonly used in research studies and clinical settings to stratify patients based on comorbidity burden, aiding in predicting outcomes, resource allocation, and treatment decision-making.

**Figure 2:**
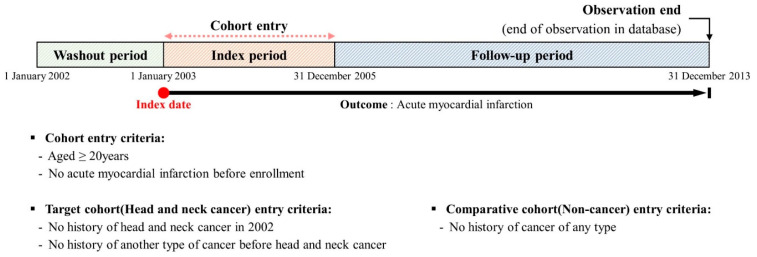
Description of the study design.

Through the implementation of a 4:1 ratio propensity score matching technique, we achieved a balanced match between the target and control cohorts. Subsequent detailed analysis confirmed that the distribution of all covariates was remarkably consistent across both groups. Table 1 shows no significant disparities in any of the independent variables examined. Furthermore, the success of the matching procedure was visually confirmed using a balance plot test, which indicated an even post-matching distribution, thus validating the effectiveness of our matching strategy. These combined results underscore the strength and appropriateness of our approach for cohort matching and highlight its efficacy in ensuring comparability between the two groups. Figure 2 details the study design, highlighting three key phases: the elimination of the initial year data (2002) to serve as a washout period for preexisting AMI cases, the baseline period for identifying HNC cases, and the follow-up period during which the study recorded the primary endpoint of AMI or death. Data from participants without AMI events up to the last follow-up were censored at that endpoint. To confirm an adequate sample size in a retrospective cohort study, it is imperative to include a minimum of 10 event instances per cohort group, which is essential for achieving statistical reliability and clinical significance of the observed outcomes [20]. In situations where achieving this ideal threshold is unfeasible, it is deemed acceptable to reduce the requisite to a minimum of seven or, in certain cases, five events per predictor variable to maintain analytical validity. This study aimed to explore the incidence and risk ratios of AMI in patients with HNC versus those without cancer by calculating the incidence of AMI per 1000 person-years from patient enrollment to their respective endpoints. We adjusted for confounding factors using multivariable-adjusted logistic regression, Cox proportional hazard, and propensity scoring. The present study used Cox proportional hazards regression to examine the effect of HNC on the likelihood of AMI, which is presented as hazard ratios (HR) with 95% confidence intervals (CI). Both the unadjusted and adjusted HRs were detailed to ensure thorough evaluation. Data analysis was performed using the R software (version 4.0.0) provided by the R Foundation for Statistical Computing in Vienna, Austria. Statistical significance was set at *p* < 0.05.

### 2.3. Ethical Considerations and Data Availability

The study was approved by the Institutional Review Board of Hallym Medical University, Chuncheon Sacred Hospital (2021-08-006), who waived the requirement for written informed consent from the participants. This exemption was granted based on the use of a cohort database lacking identifiable information, thus adhering to South Korea’s stringent privacy laws. The principal investigator received the cohort data in an anonymized format in line with the regulations of the Korean National Health Insurance Service, which stipulate that such data cannot be made public. Despite these restrictions, the integrity of our study’s findings was supported by thorough documentation within the article itself, and we conducted our research in strict compliance with the ethical standards outlined in the Declaration of Helsinki. It should be noted that while the data are not publicly available, interested parties can request access to the datasets used by contacting the study’s corresponding author under certain conditions.

## 3. Results

### 3.1. Effect of HNC on Subsequent Development of AMI

An extensive review encompassing 26,002.8 person-years in the comparative group and 5476.2 person-years in the target group was utilized to evaluate the occurrence and risk ratios of AMI throughout the study (Table 2). Our findings revealed an AMI incidence rate of 2.19 in the HNC group, in contrast to an incidence rate of 2.35 in the control group, indicating comparative incidence rates between those diagnosed with HNC and those without cancer. Moreover, the Cox regression analysis revealed no statistically significant overall risk for subsequent AMI development in HNC patients (HR: 0.93, 95% CI: 0.50–1.73) during the follow-up period. An analysis of the risk of developing AMI during the follow-up phase, categorized by the time elapsed since the diagnosis of HNC, revealed that the likelihood of AMI occurrence remained consistent with that of the control group, showing no increased risk across varying durations post-HNC diagnosis (Supplementary Table S1).

### 3.2. Evaluation of the Risk for AMI According to the Subgroups

Subsequent subgroup analyses segmented by sex revealed that the incidence of AMI in patients with HNC did not increase in either men or women (Table 3). Age-based analysis further indicated no variance in AMI risk across different age groups (Table 4), which suggests that the AMI risk in patients with HNC is independent of sex and age. Moreover, there was no significant difference in the risk of developing AMI in patients with HNC according to the comorbidity status (Table 5). To assess the AMI risk across various HNC subtypes, including the oral cavity, salivary gland, oropharynx, nasopharynx, hypopharynx, sinus tract, and larynx, we applied multivariable-adjusted logistic regression models to each subgroup. These analyses revealed that none of the HNC subtypes exhibited a statistically significant increase in the risk of AMI (Supplementary Table S2).

## 4. Discussion

The relationship between HNC and AMI is a complex and multifaceted area of medical research that is drawing attention because of its potential implications for patient care and outcomes. HNC encompasses a group of malignancies located in the oral cavity, pharynx, larynx, and other adjacent areas, whereas AMI refers to heart conditions caused by narrowed heart arteries, leading to reduced blood supply to the heart muscle and potentially a heart attack. Research on the correlation between HNC and AMI aims to understand how these diseases influence each other and identify strategies to mitigate the risk of cardiovascular disease in patients with HNC. In this population-based longitudinal study, we examined the association between HNC and the risk of AMI using a cohort database of 1,025,340 South Korean patients. Interestingly, there was no significant association between the subsequent development of AMI in HNC survivors who did not receive radiation or chemotherapy. In addition, we did not observe an increased incidence or risk of incident AMI events in any subgroup when stratified by sex, age, HNC subtype, or comorbidities.

It is well known that cardiovascular disease is associated with patients with HNC [21,22,23]. To date, several treatment-related and shared risk factors have contributed to the interconnection between HNC and ischemic cardiovascular diseases, wherein the treatment of HNC, particularly radiation therapy, can increase the risk of developing ischemic cardiovascular disease. Radiation can induce inflammation and fibrosis in blood vessels, accelerating atherosclerosis (hardening and narrowing of the arteries) and increasing the risk of coronary artery disease [9,13,15,24]. HNC and ischemic cardiovascular disease share common risk factors, including tobacco use, alcohol consumption, and, for some types of HNC, human papillomavirus infection. These shared risk factors can independently contribute to the development of both conditions [25,26,27]. However, to date, there have been no detailed investigations of the risk of ischemic events associated with HNC. Thus, this study used a matched control group drawn from individuals with no cancer diagnosis during the entire observation period. CCI was also used to control systemic diseases, such as HTN and DM, which are known risk factors for ischemic events, although we could not adjust for behavior-related risk factors, such as smoking and alcohol consumption. In addition, the present study selected a target group of patients with HNC who did not receive any radiation or chemotherapy. To our knowledge, this is the first study to analyze the effect of HNC on the subsequent development of ischemic cardiovascular disease.

Managing HNC involves a collaborative approach that involves specialists such as head and neck surgeons, radiation oncologists, hemato-oncologists, and cardio-oncologists. Neck irradiation can lead to inflammation within arteries; however, the process by which this occurs remains largely unclear. Several studies have investigated the pathophysiology of irradiation-induced vasculopathy. Oxidative stress, which is triggered by reactive oxygen species, leads to endothelial dysfunction and inflammatory responses in areas exposed to radiation [28]. Consequently, radiation therapy promotes the release of thromboxane [29], which increases the concentration of the von Willebrand factor, facilitating the adhesion of platelets to endothelial cells and increasing the likelihood of arterial thrombosis [30]. Therefore, it is necessary to regularly screen for the carotid artery status after radiation therapy in patients with HNC. Chemotherapy in patients with various types of cancers, such as HNC and breast cancer, has been associated with a heightened risk of thromboembolic events. There have also been reports of ischemic cerebrovascular accidents occurring as a consequence of chemotherapy in these patient groups [10,14,31,32]. Various factors have been proposed as contributors to thromboembolism, including tumor embolization, vasculitis, nonbacterial thrombotic endocarditis, coagulopathy due to consumption, and adverse effects associated with chemotherapy drugs [33,34,35]. Cisplatin is the main anticancer agent used in HNC and commonly causes cerebrovascular events [10]. Therefore, to accurately assess the incidence and risk of AMI due to HNC, we excluded patients who received chemotherapy or radiotherapy. Patients who underwent surgery alone were included in the study. In addition, comparisons were made after adjusting for comorbidities that are known to be risk factors for AMI, such as HTN and DM.

The incidence rate measures the occurrence of a specific event over person-years, whereas HR assesses the risk associated with a particular exposure for a specific event. If the HR is statistically significant, it implies that the observed difference in incidence rates is likely meaningful and attributable to the exposure. However, when the HR is not statistically significant, any difference in incidence rates may be more likely attributed to chance or incidental timing rather than a true effect. Thus, in the case of observing a higher incidence rate in the HNC group, our interpretation was that this increase might be a coincidence of timing rather than a definitive causal relationship. Our study also had several methodological advantages. First, it leveraged national population data to scrutinize the frequency and risk of AMI among individuals with HNC by employing propensity score matching to adjust for key confounders. This technique ensures a rigorous comparison of AMI outcomes between patients with HNC and carefully selected control subjects. Second, the study benefited from a solid framework that engaged a large patient group over an 11-year observational period. This duration permitted an in-depth exploration of how HNC diagnosis is related to the emergence of AMI over time. Third, the selection process for control participants from the database was meticulously conducted to align sociodemographic factors with those of patients with HNC with the aim of reducing surveillance bias in evaluating AMI risk. This strategy enhanced the validity of our conclusions by ensuring that the control group mirrored patients with HNC in terms of their sociodemographic attributes. Fourth, the inclusion of a one-year washout phase before the commencement of the study period aided in further strengthening our conclusions by diminishing the influence of length-time bias. Finally, our investigation meticulously examined how the risk of AMI evolved over an extended observation period. This study aimed to determine whether the heightened risk of AMI observed in patients with HNC without radiation or chemotherapy persisted over time or is a transient phenomenon. Our results consistently indicated a negligible long-term increase in AMI risk after HNC diagnosis, reinforcing the notion that the link between HNC and AMI is minimal, which suggests an inherent association rather than coincidental findings.

Nevertheless, this study had several limitations. First, the reliance on the International Classification of Diseases, Tenth Revision, and Clinical Modification diagnostic codes for disease identification rather than a detailed review of individual patient records limited the depth of analysis. This approach precludes the acquisition of detailed patient histories and pathological findings that are essential for a more nuanced understanding of the diseases being studied. As a result, critical aspects, such as the cancer stage or onset time, were not considered, diminishing the analytical precision of the study. Second, the database used in this study contained a limited range of variables that excluded potential confounders, such as family health history, smoking habits, and alcohol use. The lack of detailed data on these confounding factors introduced possible bias in the findings. The age of patients, which were presented in broad categories to maintain anonymity, further complicated precise age group matching that potentially led to additional bias. Third, the HNC cohort included patients with both squamous and non-squamous cell carcinomas, adding complexity to the analysis. These limitations underscore the need for further studies with a broader scope of variables and a prospective approach to clarify the intricate pathways connecting HNC with AMI. The retrospective nature of this study also limited direct investigation into the causal mechanisms linking HNC with AMI, highlighting the need for future research with a more inclusive dataset and a forward-looking study design to uncover the detailed pathophysiological dynamics between these conditions.

## 5. Conclusions

In conclusion, the present study showed that patients with HNC who did not receive radiation or chemotherapy did not have a higher incidence or risk of AMI events than those without cancer. The incidence and risk of AMI events did not increase, regardless of sex, age, HNC subtype, or comorbidities. We hypothesized that ischemic cardiovascular risk may not increase due to the effect of HNC itself but rather due to the effect of treatments such as radiation and chemotherapy.

## Figures and Tables

**Table 1:** Characteristics of the study subjects.

Variables	Comparison (*n* = 2976) ^1^	Head and Neck Cancer (*n* = 744)
**Sex**		
Male	1420 (47.7%)	355 (47.7%)
Female	1556 (52.3%)	389 (52.3%)
**Ages (years)**		
<45	316 (10.6%)	79 (10.6%)
45–64	1100 (37.0%)	275 (37.0%)
>64	1560 (52.4%)	390 (52.4%)
**Residence**		
Seoul ^2^	400 (13.4%)	100 (13.4%)
Second area ^3^	520 (17.5%)	130 (17.5%)
Third area ^4^	2056 (69.1%)	514 (69.1%)
**Household income**		
Low (0–30%)	776 (26.1%)	194 (26.1%)
Middle (30–70%)	940 (31.6%)	235 (31.6%)
High (70–100%)	1260 (42.3%)	315 (42.3%)
**CCI**		
0	1744 (58.6%)	436 (58.6%)
1	668 (22.4%)	167 (22.4%)
≥2	564 (19.0%)	141 (19.0%)

^1^ Comparison of subjects without cancer. ^2^ Seoul, the largest metropolitan area. ^3^ Second area, other metropolitan cities. ^4^ Third area, other areas. CCI, Charlson Comorbidity Index.

**Table 2:** Incidence and risk of an AMI event.

Variables	N	Case	Person-Years	Incidence Rate	Unadjusted HR (95% CI)	Adjusted HR (95% CI)
**AMI**						
Comparison	2976	61	26,002.8	2.35	1.00 (ref)	1.00 (ref)
Head and neck Cancer	744	12	5476.2	2.19	0.91 (0.49–1.70)	0.93 (0.50–1.73)

AMI, acute myocardial infarction; HR, hazard ratio; CI, confidence interval.

**Table 3:** The risk ratios of AMI based on sex between the comparison (non-cancer) and HNC.

Sex	Male		Female	
	Comparison	HNC	Comparison	HNC
**AMI**				
Unadjusted HR (95% CI)	1.00 (ref)	0.88 (0.37–2.09)	1.00 (ref)	0.98 (0.40–2.38)
Adjusted HR (95% CI)	1.00 (ref)	0.89 (0.37–2.13)	1.00 (ref)	0.98 (0.40–2.38)

AMI, acute myocardial infarction; HNC, head and neck cancer; HR, hazard ratio; CI, confidence interval.

**Table 4:** The risk ratios of AMI based on age group between the comparison (non-cancer) and HNC.

Ages (Years)	<45		45–64		>64	
	Comparison	HNC	Comparison	HNC	Comparison	HNC
**AMI**						
Unadjusted HR (95% CI)	1.00 (ref)	1.50 (0.16–14.45)	1.00 (ref)	1.20 (0.34–4.32)	1.00 (ref)	0.80 (0.38–1.70)
Adjusted HR (95% CI)	1.00 (ref)	1.52 (0.16–14.68)	1.00 (ref)	1.18 (0.33–4.24)	1.00 (ref)	0.82 (0.38–1.73)

AMI, acute myocardial infarction; HNC, head and neck cancer; HR, hazard ratio; CI, confidence interval.

**Table 5:** The risk ratios of AMI based on the CCI between the comparison (non-cancer) and HNC.

CCI	0		1		≥2	
	Comparison	HNC	Comparison	HNC	Comparison	HNC
**AMI**						
Unadjusted HR (95% CI)	1.00 (ref)	1.15 (0.50–2.64)	1.00 (ref)	0.28 (0.04–2.10)	1.00 (ref)	1.13 (0.37–3.40)
Adjusted HR (95% CI)	1.00 (ref)	1.15 (0.50–2.64)	1.00 (ref)	0.30 (0.04–2.26)	1.00 (ref)	1.11 (0.37–3.35)

AMI, acute myocardial infarction; CCI, Charlson Comorbidity Index; HNC, head and neck cancer; HR, hazard ratio; CI, confidence interval; HNC, head and neck cancer.

## Data Availability

The datasets generated and/or analyzed in the current study are not publicly available owing to the policy of the Korea National Health Insurance Service but are available from the corresponding author upon reasonable request.

## Supplementary Materials

The following supporting information can be downloaded at: https://www.mdpi.com/article/10.3390/cancers16101930/s1, Supplementary Table S1. Risk of an AMI event based on the time elapsed since the diagnosis of HNC; Supplementary Table S2. Incidence and risk of an AMI event according to the subtype of HNC.
