# Supplementary material for: Exploring the Link between Head and Neck Cancer and the Elevated Risk of Acute Myocardial Infarction: A National Population-Based Cohort Study
Source: Cancers (Basel). 2024 May 18;16(10):1930. doi: 10.3390/cancers16101930 (PMC11119621; doi:10.3390/cancers16101930)
Supplement: Supplementary file 1 [file cancers-16-01930-s001.zip › cancers-2989548-supplementary.pdf]

**Supplementary Table S1.** Risk of an AMI event based on the time elapsed since the diagnosis of HNC.

| Time (Year) | AMI | |
|---|---|---|
| | Unadjusted HR (95% CI) | Adjusted HR (95% CI) |
| 1 | 0.90 (0.20–4.19) | 0.91 (0.20–4.22) |
| 2 | 0.69 (0.20–2.34) | 0.70 (0.21–2.37) |
| 3 | 0.78 (0.30–2.02) | 0.78 (0.30–2.03) |
| 4 | 1.03 (0.48–2.24) | 1.04 (0.48–2.25) |
| 5 | 0.93 (0.43–1.99) | 0.94 (0.44–2.01) |
| 6 | 0.91 (0.44–1.87) | 0.92 (0.45–1.88) |
| 7 | 1.03 (0.53–1.98) | 1.03 (0.53–1.99) |
| 8 | 0.94 (0.49–1.80) | 0.94 (0.49–1.81) |
| 9 | 0.93 (0.50–1.73) | 0.94 (0.51–1.76) |
| 10 | 0.91 (0.49–1.70) | 0.93 (0.50–1.73) |

**Supplementary Table S2.** Incidence and risk of an AMI event according to the subtype of HNC.

| **Variables** | **N** | **Case** | **Person-years** | **Incidence rate** | **Unadjusted HR (95% CI)** | **Adjusted HR (95% CI)** |
|---|---|---|---|---|---|---|
| **Cancer type** | | | | | | |
| Comparison | 2976 | 61 | 26002.8 | 2.35 | 1.00 (ref) | 1.00 (ref) |
| Oral cavity | 525 | 9 | 4057.0 | 2.22 | 0.93 (0.46–1.87) | 0.90 (0.45–1.82) |
| Salivary gland | 23 | 1 | 154.4 | 6.48 | 2.59 (0.36–18.67) | 4.44 (0.58–33.72) |
| Oropharynx | 26 | 1 | 185.3 | 5.40 | 2.26 (0.31–16.32) | 2.67 (0.37–19.48) |
| Nasopharynx | 39 | 0 | 236.8 | - | 0.00 (0–Inf) | 0.00 (0–Inf) |
| Hypopharynx | 13 | 0 | 91.5 | - | 0.00 (0–Inf) | 0.00 (0–Inf) |
| Sinonasal tract | 16 | 1 | 94.7 | 10.56 | 4.23 (0.59–30.57) | 7.41 (0.98–56.10) |
| Larynx | 102 | 0 | 656.5 | - | 0.00 (0–Inf) | 0.00 (0–Inf) |

AMI, acute myocardial infarction; HNC, head and neck cancer; HR, hazard ratio; CI, confidence interval; Inf, infinite.
